# Dispersion pattern of *Mansonia* in the surroundings of the Amazon Jirau Hydroelectric Power Plant

**DOI:** 10.1038/s41598-021-03682-1

**Published:** 2021-12-20

**Authors:** Cecilia Ferreira de Mello, Jeronimo Alencar

**Affiliations:** 1 Diptera Laboratory, Oswaldo Cruz Institute (Fiocruz), Avenida Brasil 4365, Manguinhos, Rio de Janeiro, RJ 21040-360 Brazil; 2 Postgraduate Program in Animal Biology, Institute of Biology, Federal Rural University of Rio de Janeiro, BR 465 Rd, Km 7, Seropédica, RJ 23897-000 Brazil

**Keywords:** Zoology, Animal behaviour, Entomology

## Abstract

*Mansonia* spp. are voracious hematophagous mosquitoes whose mature stages usually breed in freshwater bodies containing aquatic vegetation. The reduction in water flow leads to a proliferation in aquatic plants, increasing their populations. Besides, some species are potential vectors of pathogens such as arboviruses and microfilariae. We evaluated the degree of active dispersion of females of *Mansonia* spp. in the surrounding area of the Jirau hydroelectric power plant in the Amazon, Rondônia, Brazil, using mark-release-recapture techniques. The flight behavior of the recaptured specimens was summarized with a set of average and maximum distances traveled. We show that the dispersal movement of *Mansonia* spp. is predominantly performed by random, low, and short flights, with a tendency to remain near the breeding sites in certain vegetation fragments. However, the maximum distances traveled were 2000 m from the release point for *Mansonia amazonensis* during 2018 and *Mansonia humeralis* during 2019.

## Introduction

*Mansonia* Blanchard, 1901 has 25 species in two subgenera: *Mansonioides* and *Mansonia* (Theobald, 1907). The 10 species of *Mansonioides* are found in the tropical regions of Africa and Asia. A recent review of subgenus *Mansonia*, which redescribed the males, females, and fourth instar larvae of the genus, counted 15 valid species, 12 of which were found in Brazil^1–4^.

*Mansonia* females are aggressive and voracious hematophagous mosquitoes, with preferential nocturnal activity. Although these mosquitoes mainly live in forests, they can settle in the outskirts of cities and inhabited centers when favorable conditions are present in the form of suitable breeding sites^1,3,5^.

Active immature forms of *Mansonia* have a spiracular apparatus adapted to piercing submerged vegetation, latching on to aquatic plants, and extracting oxygen from their airy parenchyma^5^. The high abundance of aquatic plants, linked to a reduction in the flow of water with or without an increase of organic matter, can therefore facilitate the proliferation of these mosquitoes.

*Mansonia* species are pests that represent a danger to human life and livestock in certain regions^3^, rendering some places unsuitable for living or livestock farming due to their aggressive hematophagous behavior.

*Mansonia indubitans* Dyar & Shannon, 1925 and *Ma. titillans* (Walker, 1848) have already been found to be infected with arboviruses, including ones that cause encephalitis, making them potential vectors for these viruses^6^.

Studying the Culicidae fauna is essential in areas where human intervention causes environmental change. Environmental impacts can cause the population density of some species to increase, posing a risk to human health. Mosquito species with typically wild habits often adapt to live in urban areas^7,8^.

The mark-release-recapture is a surveying method that allows researchers to estimate the density, survival, and dispersion of animal populations. This method has been applied to populations of biological vectors of infectious agents for assessing vectorial capacity and estimating the flight radius of mosquito populations^9–11^.

The dispersal of insect vectors is particularly important for epidemiology since dispersal is a key factor in the progression of plant disease outbreaks and the population dynamics of vector and pest arthropods^12^. The present study aims to analyze the movement patterns of *Mansonia* spp. in the surrounding area of the Jirau hydroelectric power plant (HPP) in Rondônia state, in the Brazilian Amazon.

## Material and methods

### Ethical considerations

The permanent license number 58855 for collecting, capturing, and transporting biological material was granted by the Authorization and Information System in Biodiversity (SISBIO)—Chico Mendes Institute for Biodiversity Conservation.

### Study area

The sampling planning considered exploring the site and its surroundings, choosing the most representative capture points and the most efficient equipment, as well as selecting the methods of sample preservation.

The study was carried out in the surrounding area of the Jirau HPP, located in the Amazon Forest biome. The Jirau HPP (9° 16′ 16.8″ S; 64° 38′ 25.9″ W) is located near the city of Porto Velho. The sampling area near the release and recapture points had larval habitat with a high concentration of aquatic vegetation of the following plant species: *Eichornia crassipes* (Mart.) Solms-Laubach, *Eichornia azurea* (Sw.) Kunth, *Ceratopteris pteridoides* (Hook.) Hieron, *Pistia* sp., *Salvinia* sp., *Paspalum* sp. *Hymenachne* sp., *Oxicarium cubensis*, *Ludwigia helminthorriza*, *Phyllantus fluitans*, *Eleocharis* sp., and grasses. The respective larval habitats were characterized by a team specializing in macrophytes and another in entomology. Subsequently, they were confirmed by in-situ observation according to the descriptions of Sioli^13^. The region’s Köppen climate classification is tropical (type Aw), with a distinguishable dry season in winter from May to October (July is the driest month) and a rainy season in the summer from November to April. The average annual temperature is 25.6 °C, with monthly averages as low as 16 °C in the coldest month and over 34 °C in the warmest ones. Precipitation is over 2000 mm per year^14^.

**Figure 1:**
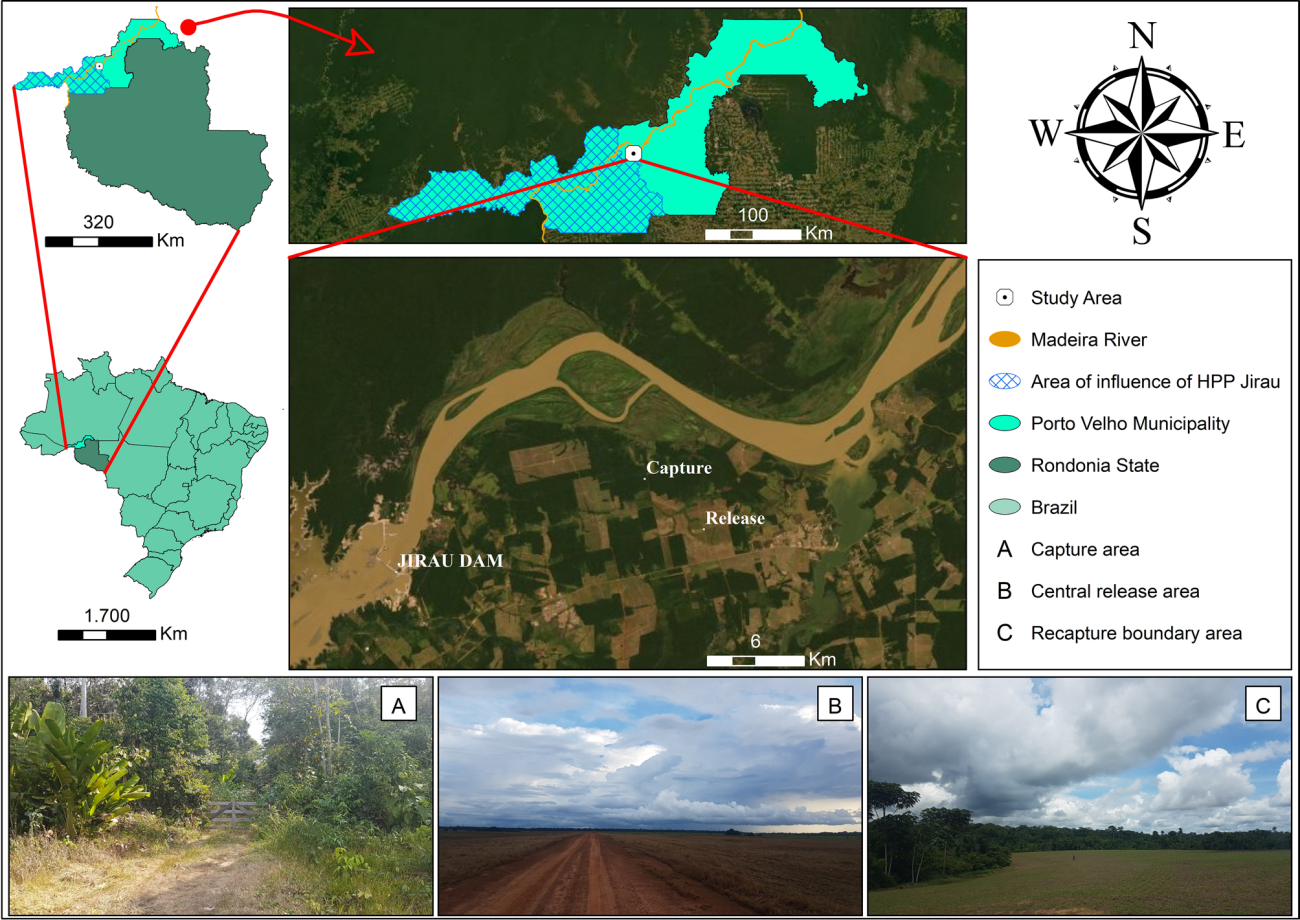
Location of the study area and sampling and release sites in the surrounding area of the Amazon Jirau Hydroelectric Power Plant (HPP-Jirau), Jaci-Paraná district, Porto Velho, Rondônia state, Brazil. *Source*: Google Earth®, Maxar Tecnologies® satellite image/Pass date: June 2019.

The capture, release, and recapture points were located in Jaci-Paraná, a district of Porto Velho, the capital of Rondônia. The specimens were captured in fragments of the closed forest (9° 13′ 02.1″ S 64° 30′ 06.6″ W) and released and recaptured in an open field (9° 14′ 50.3″ S 64° 28′ 06.5″ W). An aerial image was used to delineate the mark-release-recapture (MRR) experiment area. This image was georeferenced using a Garmin® GPS unit (Fig. 1).

### Specimen capture method

Sampling was carried out in May, July, October, and December 2018, and in April, July, September, and November 2019. The captures were conducted between 6:00 p.m. and 8:00 p.m. on three alternate days using Shannon light traps and electric aspirators used similarly to a manual suction tube to optimize the sampling effort. A 12-V rechargeable battery operated the electric aspirator. The simultaneous use of two devices makes them more efficient at capturing mosquitoes in areas with a high density of adults, aspirating inactive specimens in the Shannon trap and active ones in flight.

Additionally, we used the Castro catcher to capture samples, which was used for five minutes at intervals of 30 min, totaling 20 min. The purpose was to relate the species captured in the electric aspirator occurring during the sampling since this collector ensures greater preservation of morphological and anatomical characteristics of the specimens, leading consequently to greater reliability in identification. These specimens were confined in cages and not tagged or released and therefore sacrificed and placed in conical tubes until identification. Meanwhile, the specimens captured with the electric aspirator were kept in a polyvinyl chloride tube measuring 100 mm in diameter and 30 cm in length, which was changed every 5 min to keep the specimens alive until they were released. These tubes were closed with a plastic cap at one end and kept in a moist chamber until their release. Finally, all specimens were counted. The sampling effort with Castro catcher and electric aspirator were as follows: Sampling 1—(5.6%; 94.4%), Sampling 2—(7.2%; 92.8%), Sampling 3—(8.4%; 91.6%), Sampling 4—(17.5%; 82.5%), Sampling 5—(3.4%; 96.6%), Sampling 6—(5.6%; 92.8%), Sampling 7—(5.6%; 93.6%), Sampling 8—(5.6%; 94.4%), respectively.

### Specimen mark and release method

Specimens were marked with fluorescent powder (BioQuip®) about an hour before the time of release and then released approximately two hours after capture on the same night in the study area. The fluorescent powder was sprayed onto the specimens using a small hand pump to mark them.

### Specimen recapture method

**Figure 2:**
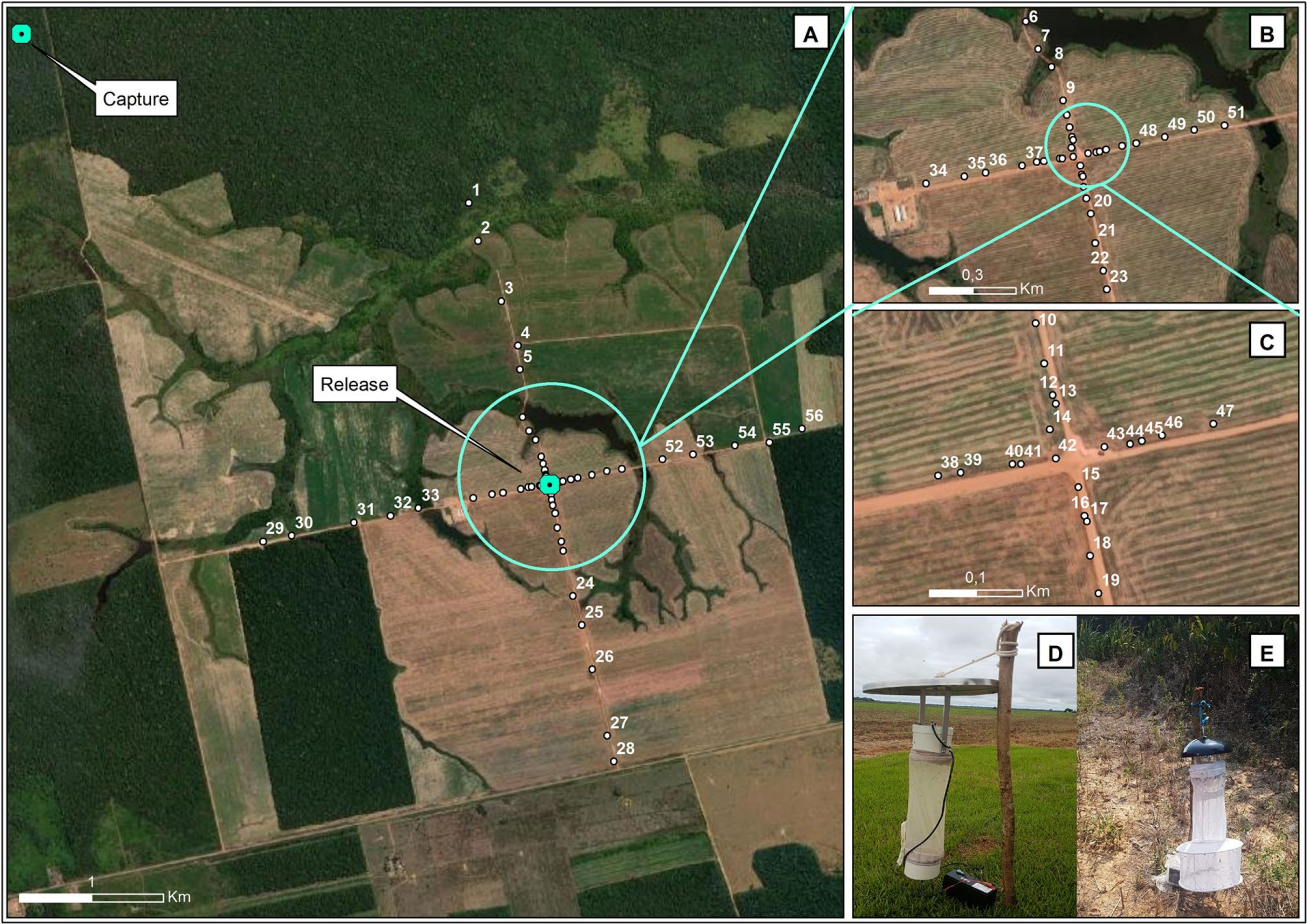
Sample design of the launch sites in the surrounding area of the Amazon Jirau Hydroelectric Power Plant (UHE-Jirau), Jaci-Paraná district, Porto Velho, Rondônia state, Brazil. (**A**) 1, 29 (Mf-60), 28, 56 (CDC) = 2000 m; 2, 30 (CDC), 27, 55 (Mf-60) = 1800 m; 3, 31 (Mf-60), 26, 54 (CDC) = 1300 m; 4, 32 (CDC), 25, 53 (Mf-60) = 1000 m; 5, 33 (Mf-60), 24, 52 (CDC) = 800 m; (**B**) 6, 34 (CDC), 23, 51 (Mf-60) = 500 m; 7, 35 (Mf-60), 22, 50 (CDC) = 400 m; 8, 36 (CDC), 21, 49 (Mf-60) = 300 m; 9, 37 (Mf-60), 20, 48 (CDC) = 200 m; (**C**) 10, 38 (CDC), 19, 47 (Mf-60) = 150 m; 11, 39 (Mf-60), 18, 46 (CDC) = 100 m; 12, 40 (CDC), 17, 45 (Mf-60) = 70 m; 13, 41 (Mf-60), 16, 44 (CDC) = 60 m; 14, 42 (CDC), 15, 43 (Mf-60) = 30 m; (**D**) CDC-type traps; (**E**) MF-60 light trap with CO_2_. *Source*: Google Earth®, Maxar Tecnologies® satellite image/Pass date: June 2019.

Recaptures were carried out with CDC and MF 60® light traps. Initially, wooden stakes of ≈ 1 m were driven into the ground so as to suspend the light traps. Then, 28 CDC-type traps and 28 MF60 traps were installed at distances between 30 m and 2000 m to the north, south, east, or west of the release site. The capture and recapture points remained in the same place throughout the sampling season; traps were monitored and changed every 24 h (Fig. 2). The recaptures in the sample area extended over six subsequent days.

### Identification

Species of *Mansonia* were identified by direct observation of morphological characters under a stereomicroscope (Zeiss®) and an optical microscope with ultraviolet light (Nikon®). We used dichotomous keys^15^, to consult the species descriptions and associated biological characteristics and behaviors. Aiming to ensure the specific identification of *Mansonia* females, the arrangement pattern of the spines on the last abdominal tergite was compared. All specimens from each sample period had their eighth abdominal segment sectioned. The structures were washed in distilled water and neutral detergent to loosen all scales from the structure. Then, the structures were dehydrated with 70% to 95% alcoholic series in absolute alcohol. The eighth abdominal segment was mounted dorsally with Canada balsam, between slide and coverslip. The specimens were subsequently deposited in the Entomological Collection of the Instituto Oswaldo Cruz, under the title “Amazon Collection, UHE-JIRAU”.

The abbreviations of genera and subgenera followed the norms suggested by Reinert^16^, modified according to the proposed nomenclature for mosquitoes.

A set of dispersion measures composed of the mean (MDT) and maximum (MAX) distance traveled were used to summarize the flight behavior of the recaptured culicids^17,18^.

## Results

A total of 38,113 females of *Mansonia* spp. were captured, of which 26,677 (70%) were marked and released, and 11,436 (30%) were preserved for species identification. This allowed for an approximate representation of the species captured, marked, and released in each experiment.

During the dry, rainy, and transition periods of 2018 and 2019, a total of 169 recaptured specimens were recorded over the eight sampling campaigns (Table 1). Recaptures are represented by eight species of the genus *Mansonia*, with a different number of specimens recaptured in each seasonal period, showing distinct trends in species abundance and dispersion from the release point (Table 2).

**Table 1:** Distribution of the number of estimated specimens captured, marked, released, and recaptured in the surrounding area of the Amazon Jirau (HPP-Jirau) hydroelectric power plant, Rondônia state, Brazil, from 2018 to 2019.

Year	Sampling	Season	Captured	Marked and released	Migration distance (M)	Marked specimens found	Total
2018	1	Transition	8175	5722	1000	1	3
					100	2	
	2	Dry	5981	4187	200	10	24
					30	5	
					60	7	
					70	2	
	3	Rainy	3565	2495	150	6	17
					200	1	
					300	7	
					400	2	
					500	1	
	4	Rainy	394	276	2000	1	1
2019	5	Rainy	2805	1963	1300	6	6
	6	Dry	5142	3599	800	11	32
					1300	15	
					1800	6	
	7	Transition	6026	4218	100	3	29
					300	2	
					500	2	
					1000	18	
					2000	4	
	8	Rainy	6025	4217	300	20	57
					500	18	
					1000	2	
					2000	17	
	Total		38,113	26,677		169	169

**Table 2:** Flight distance traveled by marked, released and recaptured *Mansonia* spp. in the surrounding area of the Amazon Jirau (HPP-Jirau) hydroelectric power plant. (Samplings: 1 to 8).

Species	2018		2019	
	ADT (m)	MAX (m)	ADT (m)	MAX (m)
*Mansonia amazonensis*	390	2000	1000	1800
*Mansonia flaveola*	168.57	400	–	–
*Mansonia humeralis*	253	2000	1158.8	2000
*Mansonia iguassuensis*	–	–	750	1300
*Mansonia indubitans*	194.17	300	–	–
*Mansonia pseudotitillans*	300	300	625	800
*Mansonia titillans*	383.33	1000	1133.3	1300
*Mansonia wilsoni*	150	150	1300	1300

*ADT* Average distance traveled, *MAX* Maximum distance traveled.

Two species were recaptured at the maximum distance of 2000 m from the release site: Ma. Amazonensis (Theobald, 1901) and Ma. humeralis Dyar & Knab (Table 2).

The largest number of recaptured individuals belonged to *Ma. humeralis* (47 events), *Ma. titillans* (45 events), and *Ma. amazonensis* (42 events). A smaller number of recaptures were recorded for *Ma. pseudotitillans* (Theobald, 1901) (five events) and *Ma. wilsoni* (Barreto & Coutinho, 1944) (three events).

Of the 12,680 estimated specimens marked and released in 2018, 45 individuals (0.35%) were recaptured. These were subsequently identified as *Ma. indubitans* (27%), *Ma. humeralis* (24%), *Ma. flaveola* (16%), *Ma. amazonensis* (16%), *Ma. titillans* (13%), *Ma. wilsoni* (2%), and *Ma. pseudotitillans* (2%).

In 2019, 124 individuals (0.88%) of the 13,997 specimens marked and released were recaptured; these were identified as *Ma. titillans* (31%), *Ma. humeralis* (29%), *Ma. amazonensis* (28%), *Ma. iguassuensis* (6%), *Ma. pseudotitillans* (3%), and *Ma. wilsoni* (2%).

The highest number of recaptures of *Mansonia* spp. occurred at a distance of 300 m (N = 29) from the release site. The maximum dispersal distance of 2000 m was observed during sampling periods 4, 7, and 8 for all taxa. Sampling period 8, performed in the rainy season, had the highest number of recaptured specimens (N = 57) (Table 1).

Throughout the study period, specimens tagged with fluorescent powder were found in traps 25 times. Sampling periods 3 and 7 had traps with tagged mosquitoes at five different dispersion distances (Table 1). Tagged mosquitoes were found in traps at distances of 300, 500, 1000, and 2000 m in three sampling periods (Table 1).

## Discussion

The movement of *Mansonia* species observed in the surrounding area of the Jirau HPP was consistent with dispersion patterns observed by a previous study^19^. The authors analyzed the dispersal behavior of three mosquito species in two habitats. They found that 70% of the population of *Ma. annulata* Leicester, 1908 dispersed within 0 m in one site and 250 m in the second. These figures were 150 and 700 m for *Ma. uniformis* (Theobald, 1901) and 150 and 720 m for *Ma. indiana* Edwards, 1930. A maximum flight range of 1700 m was found for *Ma. uniformis*.

Light traps were observed to be effective in specimen recapture. A high density of *Mansonia* spp. was observed over the study period, with dispersion distances from the release point ranging between 30 and 2000 m.

Ivanova-Kazas^20^ reported that mosquitoes move from their breeding sites when searching for a blood-feeding source and then return to their breeding sites for oviposition. Displacement movements from breeding sites to human dwellings were also observed for *Anopheles maculipennis* Meigen, 1818. These movements seem to be influenced by the topography and prevailing winds that carry attractive odors*. Mansonia perturbans* (Walker) also shows upward and downward displacements from the forest canopy at dusk and dawn^21^.

The vegetation type that makes up the background landscape of the study area, with the formation of forest mosaics in the Amazon, favors the outward movement of adults. The occurrence of forests in the vicinity of the experimental area inhibits the movement of specimens in this direction. In addition, the high availability of food sources beyond the boundary of the experimental area changes the return rates of individuals. Thus, the specimens of *Mansonia* spp. may disperse more than 2 km from the adult’s point of emergence, with a slight tendency to remain in the vegetation patch under stable conditions, i.e., in preserved environments with nearby food sources.

Gorayeb and Ribeiro^22^ studied the Tabanidae fauna from the eastern Amazon to define its species displacement autonomy. They found that the disparity in the number of species captured was influenced by seasonality. In addition, they point to intrinsic mechanisms of tabanid species in terms of finding and attacking hosts, which are clearly different over small and large distances. They also emphasize that wind is unlikely to play a role in local tabanid displacement behavior in searching for horses as blood-feeding sources.

Few studies have explored the flight intervals in *Mansonia* species. Wharton^23^ reported that *Ma. bonneae* Edwards, 1930 and *Ma. dives* (Schiner, 1868) can disperse the considerable distances of 1.6 to 3.2 km in the forest environment when searching for hosts as blood-feeding sources. In this sense, the depletion of food sources can change the population dynamics of *Mansonia* spp. by widening the dispersion range of the taxa through the search for food in other ecological niches.

The number of recaptured specimens that we recorded is consistent with other similar studies on mosquitoes^24–27^. The results from our eight recapture reference samples therefore within the expected ranges for our experimental design.

Similar observations were made in a study of *Ma. uniformis*, a species with low displacement^19^. This was also confirmed by Bailey and Gould^28^ using CDC light traps, demonstrating that the marked individuals did not fly more than 375 m from the release point.

The dispersal behavior of immature and adult specimens is an intrinsic characteristic of all species. Taxa disperse spontaneously, stimulated by several factors, and the movements occur due to the natural ability of subsistence related to the physiological needs of each species^29^.

We conclude that the dispersal movement of *Mansonia* spp. is predominantly performed by random, low (**≅ **1 m high), and short flights maintaining a home range within a radius of approximately 30 to 100 m from the adults’ point of emergence, showing a tendency to remain near the breeding sites in certain fragments of vegetation. Even so, our observations confirm that specimens of *Mansonia* spp. are capable of covering distances greater than 2 km from the adults’ emergence point, despite their general tendency to remain in the same vegetation patch under stable conditions of food sources and environmental preservation.
